# A simple APACHE IV risk dynamic nomogram that incorporates early admitted lactate for the initial assessment of 28-day mortality in critically ill patients with acute myocardial infarction

**DOI:** 10.1186/s12872-022-02960-8

**Published:** 2022-11-24

**Authors:** Jikai Song, Tianhang Yu, Qiqi Yan, Liuyang Wu, Sujing Li, Lihong Wang

**Affiliations:** 1 Zhejiang Provincial People’s Hospital, Qingdao University, Hangzhou, Zhejiang Province China; 2 North China University of Science and Technology, Tangshan, Hebei Province China

**Keywords:** APACHE IV, Nomogram, Acute myocardial infarction, eICU database, 28-day mortality

## Abstract

**Background:**

Early risk stratification is important for patients with acute myocardial infarction (AMI). We aimed to develop a simple APACHE IV dynamic nomogram, combined with easily available clinical parameters within 24 h of admission, thus improving its predictive power to assess the risk of mortality at 28 days.

**Methods:**

Clinical information on AMI patients was extracted from the eICU database v2.0. A preliminary XGBoost examination of the degree of association between all variables in the database and 28-day mortality was conducted. Univariate and multivariate logistic regression analysis were used to perform screening of variables. Based on the multifactorial analysis, a dynamic nomogram predicting 28-day mortality in these patients was developed. To cope with missing data in records with missing variables, we applied the multiple imputation method. Predictive models are evaluated in three main areas, namely discrimination, calibration, and clinical validity. The discrimination is mainly represented by the area under the receiver operating characteristic curve (AUC), net reclassification improvement (NRI) and integrated discrimination improvement (IDI). Calibration is represented by the calibration plot. Clinical validity is represented by the decision curve analysis (DCA) curve.

**Results:**

A total of 504 people were included in the study. All 504 people were used to build the predictive model, and the internal validation model used a 500-bootstrap method. Multivariate analysis showed that four variables, APACHE IV, the first sample of admission lactate, prior atrial fibrillation (AF), and gender, were included in the nomogram as independent predictors of 28-day mortality in AMI. The prediction model had an AUC of 0.819 (95%CI 0.770–0.868) whereas the internal validation model had an AUC of 0.814 (95%CI 0.765–0.860). Calibration and DCA curves indicated that the dynamic nomogram in this study were reflective of real-world conditions and could be applied clinically. The predictive model composed of these four variables outperformed a single APACHE IV in terms of NRI and IDI. The NRI was 16.4% (95% CI: 6.1–26.8%; *p* = 0.0019) and the IDI was 16.4% (95% CI: 6.0–26.8%; *p* = 0.0020). Lactate accounted for nearly half of the total NRI, which showed that lactate was the most important of the other three variables.

**Conclusion:**

The prediction model constructed by APACHE IV in combination with the first sample of admission lactate, prior AF, and gender outperformed the APACHE IV scoring system alone in predicting 28-day mortality in AMI. The prediction dynamic nomogram model was published via a website app, allowing clinicians to improve the predictive efficacy of the APACHE IV score by 16.4% in less than 1 min.

**Supplementary Information:**

The online version contains supplementary material available at 10.1186/s12872-022-02960-8.

## Introduction

Acute myocardial infarction (AMI) is the death or necrosis of myocardial cells caused by the occlusion of a coronary artery and is the most serious subtype of coronary heart disease [1]. In recent years, as AMI treatment options have improved, inpatient mortality rates have declined year on year, ranging from 2.5 to 8% [2–4]. However, the high death toll associated with the high incidence of AMI continues to impose a significant medical and psychological burden on the entire world [5, 6]. Moreover, a large retrospective cohort research of elderly AMI patients revealed that those admitted to the intensive care unit (ICU) have a much higher hospital mortality rate than patients treated to the general hospital (14.3% vs 8.3%) [7]. However, there are very few early risk prediction tools for patients with AMI based on basic bedside clinical indications. The use of an early warning tool based on data from patients with cardiovascular disease to objectively assess the risk of death or serious complications can help clinical staff to identify potential risks and intervene as early as possible, thereby reducing the risk of a patient's condition progressing to critical illness. Acute Physiology and Chronic Health Evaluation (APACHE) IV can document each new ICU patient's worst parameter value and prior medical history within the first 24 h of hospitalization [8]. APACHE IV, released in 2005, predicts the length of stay in the ICU and the risk of in-hospital death with greater accuracy than the previous three versions [9]. The APACHE IV score is an internationally accepted, non-specific method of evaluating critical illnesses, focusing on the whole body, which is simple, objective, and reliable. However, studies on the use of the APACHE IV system in cardiovascular disease are scarce, especially in patients with AMI. APACHE IV has some usefulness for forecasting cardiovascular mortality in patients with cardiovascular illness, but additional refinement is required [10].

The main mortality prediction tools for patients with AMI are the Thrombolysis in Myocardial Infarction (TIMI) score and the Global Registry of Acute Cardiac Events (GRACE) score [11–13]. These prediction tools enable risk categorization and identification of high-risk events. However, the limitations and drawbacks of these scoring tools should not be ignored. To begin with, the TIMI risk score is primarily used in ST-elevation myocardial infarction (STEMI) and has been shown to be inefficient in non-STEMI (NSTEMI). What’s more, the GRACE score fails to cover indicators such as coronary risk factors and the patient's previous cardiovascular history, which may have an impact on the prognosis of patients with AMI. This therefore affects the accuracy of these scoring systems in predicting early admission of AMI patients [14]. In 2019, Jentzer et al. from the Mayo Clinic developed a risk score called M-CARS, designed specifically for mortality risk prediction in unselected cardiac intensive care unit (CICU) patients [15]. The M-CARS allows for earlier prediction of patient mortality by using seven variables early in the CICU admission. However, M-CARS is a predictive model constructed using comprehensive data from all CICU patients, and its predictive efficacy for mortality in AMI patients is not yet known.

In recent years, a significant corpus of research has focused on laboratory indicators for predicting disease prognosis. Several recent studies have shown that lactate level is an independent prognostic factor that can aid in the identification of high-risk patients [16–18]. Baysan et al. showed that in patients with sepsis, lactate within 24 h of admission increased the predictive efficacy of APACHE IV [19]. Additionally, a recent basic study demonstrated that changes in the pyruvate-lactate axis are a precursor to cardiomyocyte hypertrophy and failure [20]. Cardiomyocyte failure is frequently a very strong predictor of death in people with AMI. Here, we sought to investigate whether lactate within 24 h of admission had a prognostic impact on 28-day mortality in patients with AMI and, if so, to design a development and validation APACHE IV nomogram incorporating lactate for the early evaluation of patients with AMI. In parallel, a web app will be developed to reduce the burden of clinical applications.

## Methods

The methods provided in this article complied with the transparent reporting of a multivariable prediction model for individual prognosis or diagnosis (TRIPOD) statement [21].

### Source of data

Data was collected from the eICU Collaborative Research Database v2.0. Throughout 2014 and 2015, eICU-CRD included 200,859 ICU admissions for 139,367 patients from 208 hospitals across the United States [22]. Hourly physiological readings from records of demographic features, the severity of sickness measures, diagnosis, therapy, and other clinical data collected during normal medical care were included in the eICU database.

The databases were built in accordance with the Massachusetts Institute of Technology's institutional review board's ethical guidelines (Cambridge, MA, USA). As a result, consent for the original data collection, which included data for this study, was acquired. All the data in this article was sourced by author Song, who completed the National Institutes of Health's training program and was granted admittance (certification number: 42287940).

### Participants and design

**Fig. 1:**
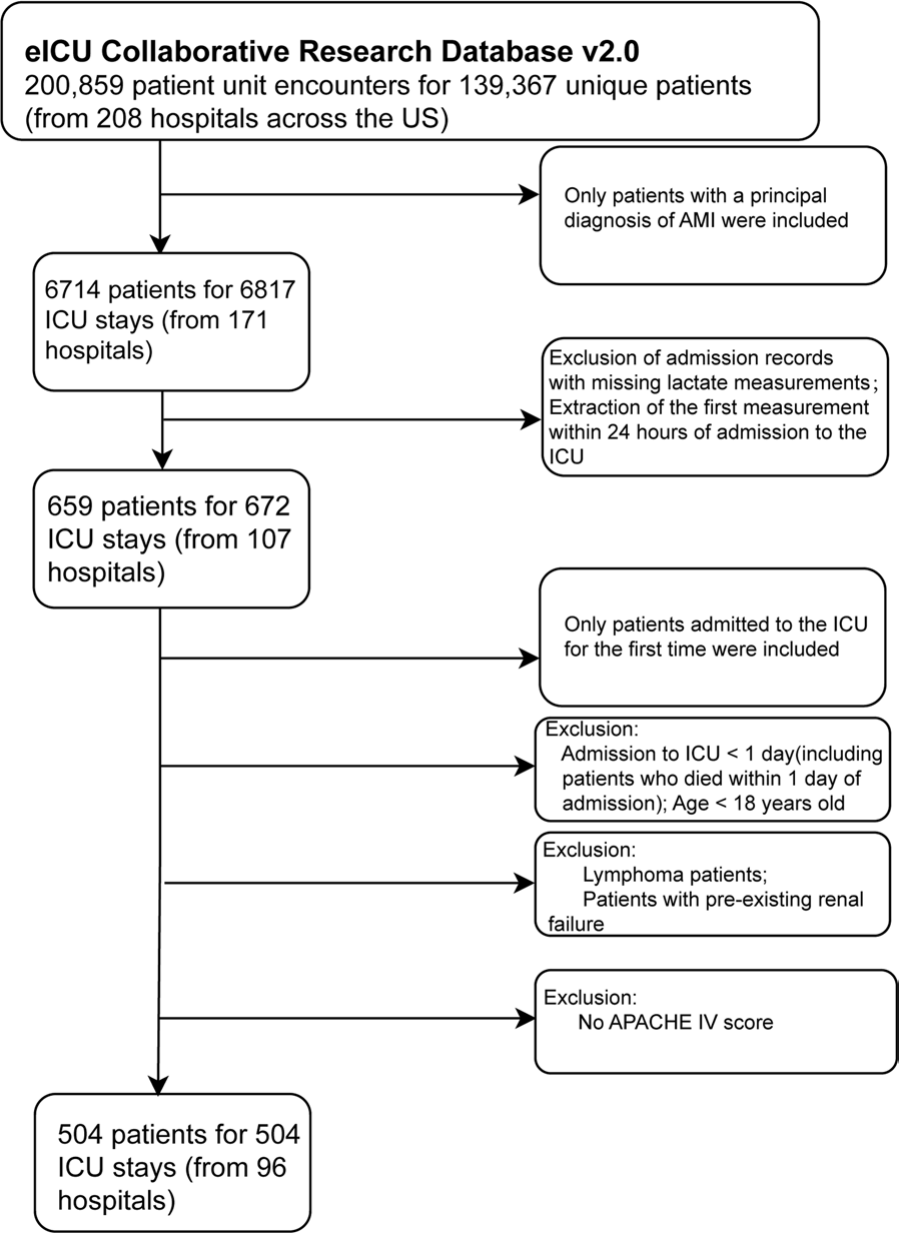
Selection of study patients

AMI was coded by trained eICU clinicians using the diagnosis system of APACHE IV in the eICU database v2.0. We selected the AMI patients in the table of APACHEPredVar. The inclusion/exclusion criteria for the process of participants are presented in Fig. 1. Only patients who were first admitted to the intensive care unit (ICU) and diagnosed with AMI were included in this study, out of the 139,367 individuals in the eICU database. The exclusion criteria were as follows: (1) admission records with missing lactate measurements; (2) admission to ICU less than 1 day (including patients who died within 1 day of admission); (3) age less than 18 years old; (4) patients with lymphoma; (5) patients with pre-existing renal failure; and (6) no APACHE IV score.

Based on these inclusion and exclusion criteria, 504 patients were included in the study. An initial screening of the importance of all variables related to 28-day mortality was carried out by XGBoost, which is a new machine learning method [23]. Due to our limited sample size, we used all 504 people to construct the prediction model according to the recommendation of the TRIPOD guide, and the validation of the model was performed internally using the bootstrap method [24]. In addition, a brief comparison with the M-CARS model was performed after the full model was constructed [15]. To avoid the reduced statistical test efficacy and bias associated with direct exclusion of missing values, multiple interpolation (MI) was used to estimate the missing values [25]. Five sets of data were generated based on the SAS chain equation [26, 27]. These five sets of data were later combined for analysis [28]. The prediction model will be displayed as a nomogram, and a web calculator to display the dynamic nomogram will be constructed. Finally, the applicability of the model was explored by comparing the baseline disease of patients admitted with or without lactate measurement in the eICU database of 6817 AMI patients.

### Clinical outcomes definitions

The study's end point was 28-day mortality, which was defined as death occurring within 28 days of ICU admission.

### Data extraction

The clinical data of each patient was collected from the eICU database using Empowerstats 3.0, which included laboratory test results, demographic information, vital signs, comorbidities, APACHE IV score, Braden skin score, AMI-location and pertinent past medical history. Laboratory tests included hemoglobin, glucose, creatinine, blood urea nitrogen (BUN), lactate, etc. Demographic parameters mainly refer to age, gender, height, and weight. Vital signs included systolic blood pressure (SBP), diastolic blood pressure (DBP), heart rate (HR), respiratory rate (RR), and percutaneous oxygen saturation (SaO_2_). Comorbidities included hepatic failure, cirrhosis, and diabetes. Pertinent past medical history included atrial fibrillation (AF), stroke, hypertension, myocardial infarction in the past 6 months (MIDUR), congestive heart failure (CHF), percutaneous coronary intervention within 24 h (PCI), thrombolysis within 24 h, coronary artery bypass grafting (CABG). The lactate in AMI patients was taken from arterial blood gas values and was measured at least once within the first 24 h. However, some patients were tested up to seven times. The rest of the laboratory tests, vital sign tests, etc., were similar. The aim of our study was to be able to predict AMI 28-day mortality at an early stage after admission, while reducing the workload of clinical staff. Therefore, the admission value of all laboratory values, vital signs, and clinical measurements was used, defined as the first value recorded after ICU admission. Shock, cardiac arrest, and respiratory failure were other diagnoses that were combined at the time of admission for AMI patients and were likewise collected.

### Statistical analysis

The Kruskal–Wallis test was used to test hypotheses on continuous variables, which were expressed as mean (standard deviation) or median (interquartile range [IQR]). Categorical variables were reported as numbers (percentages) and examined using chi-squared or Fisher's exact tests, depending on the situation.

A preliminary XGBoost examination of the degree of association between all variables in the database and 28-day mortality was conducted using the XGBoost R package [29]. We used XGBoost [30] to initially screen predictors mainly for the following reasons: (1) XGBoost techniques have their own unique advantages, which do not have high requirements on the original data, such as whether the independent variables are normally distributed, whether the variables are independent of each other, whether the variables are continuous or discrete, etc., and have strong self-organization and self-adaptive capabilities. (2) For samples with missing feature values, XGBoost can automatically learn its split direction and interpolate. (3) Overfitting can be avoided with XGBoost by using cross-validation and stopping the tree construction early.

Univariate and multivariate logistic regression models were used to analyze risk factors. In the multivariate logistic regression analysis, variables that showed statistical significance (*p* < 0.05) in the univariate analysis were included, and the backward stepwise method by generalized linear model was used to select the variables that were eventually included in the model (variables included in the APACHE IV score were excluded). To measure the effect of missing data on variable screening, we employed multiple imputation in the R MI technique, which was based on 5 replications and a chained equation approach method [31]. Additionally, we ran a multicollinearity check (VIF step screening methods) based on these probable variables before constructing the predicted nomogram. Ultimately, the Nomogram was constructed [32, 33], and the website APP of dynamic nomogram was further developed to improve its clinical utility [34]. The nomogram is designed to provide clinically relevant prognostic models as well as to provide disease-specific characteristics that can be measured specific to the individual patient. To date, the nomogram has been widely used to predict the long-term survival status of cancer patients [32, 35]. To our knowledge, the nomogram is also a potentially ideal model for predicting mortality in patients with AMI.

To assess the model's discrimination performance, the area under the curve (AUC) was determined, and a 500 bootstrap approach was used for internal validation. Enrolled patients were randomly separated into two groups for further validation: 50% for training and 50% for validating [36]. The model's clinical utility was assessed via decision curve analysis (DCA), which quantified the net benefits at various threshold probabilities [37]. Calibration plot to evaluate the accuracy of risk models in predicting the probability of future events [38]. To measure the improvement in predictive accuracy attained by adding new variables to the variable of the APACHE IV score, the net reclassification improvement (NRI) and integrated discrimination improvement (IDI) were calculated. Finally, the M-CARS prediction model was used in AMI patients and its predictive efficacy was compared with that of our constructed full model in terms of AUC, DCA, NRI, and IDI. All the statistical analysis was performed using R software version 4.0.0 (http://www.r-project.org) and the Empower Stats (www.empowerstats.com, X&Y solutions, Inc. Boston MA). Two-tailed significance with *p* < 0.05 was considered statistically significant.

## Results

### Study population

After the exclusion criteria were implemented, 504 patients remained in the trial (from 96 hospitals across the US) (Fig. 1). Table 1 presents the patient characteristics of the predictive cohort. Of the 504 patients in the predictive cohort, 86 (17.1%) died within 28 days. Of those 504 patients, 86 (17.1%) were female. The mean (SD) age was 67.75 (12.51) years and 73.88 (11.27) without or with 28-day mortality, respectively. The median (IQR) APACHE IV score and median (IQR) early lactate within 28-day mortality were 72.50 (61.00–90.00) and 2.45 (1.70–5.50). According to the information connected to a patient's relevant past medical history, 69 (80.23%) of the 86 patients who died within 28 days in the hospital had no history of AF, while 17 (19.77%) did. In addition, the largest patient populations in AMI-location were non-Q AMI and anterior AMI. As seen in Additional file 1: Table S1, 17.41% of patients with non-Q AMI and 16.67% of patients with anterior AMI died within 28 days, with a p value of 0.874. However, non-Q AMI had an older age, a higher proportion of prior AF, prior CHF, and prior CABG, and a lower proportion of PCI within 24 h and thrombolytics within 24 h compared with anterior AMI.

**Table 1:** Baseline demographic and clinical characteristics of the study participants

Variable	28-day mortality		*P-*value
	No (n = 418)	Yes (n = 86)	
Baseline characteristics			
Age (years)	67.75 (12.51)	73.88 (11.27)	< 0.001
Gender, n (%)			0.002
Male	261 (62.44%)	38 (44.19%)	
Female	157 (37.56%)	48 (55.81%)	
Height (m)	1.70 (0.11)	1.66 (0.10)	0.002
Weight (Kg)	83.21 (20.57)	75.39 (21.88)	0.002
Distribution of mortality days and length of stays	7.47 (4.60–12.02)	3.75 (1.87–7.44)	< 0.001
Laboratory test			
Hemoglobin (g/dl)	12.10 (2.18)	11.56 (2.34)	0.042
Glucose (mmol/L)	7.90 (6.40–10.70)	8.40 (6.90–13.57)	0.077
Creatinine (mg/dl)	1.10 (0.82–1.55)	1.33 (0.90–1.99)	0.006
BUN (mg/dl)	20.00 (15.00–31.00)	25.50 (17.00–37.00)	< 0.001
Lactate (mmol/L)	1.57 (1.10–2.40)	2.45 (1.70–5.50)	< 0.001
PH	7.36 (0.10)	7.33 (0.12)	0.026
AG	13.19 (5.20)	13.96 (5.47)	0.216
RDW	14.44 (1.87)	14.67 (1.91)	0.234
Scoring systems			
APACHE IV score	54.00 (41.00–70.00)	72.50 (61.00–90.00)	< 0.001
Braden skin score	16.43 (3.37)	16.23 (3.20)	0.586
Vital signs			
SBP (mmHg)	120.36 (23.38)	114.68 (23.97)	0.051
DBP (mmHg)	70.34 (15.20)	65.57 (15.21)	0.011
RR (beats/minute)	20.09 (5.29)	21.90 (6.09)	0.006
Comorbidities, n (%)			
Diabetes			0.093
No	293 (70.10%)	68 (79.07%)	
Yes	125 (29.90%)	18 (20.93%)	
AMI-location			0.715
Non-Q	185 (44.26%)	39 (45.35%)	
Anterior	75 (17.94%)	15 (17.44%)	
Anterolateral	29 (6.94%)	7 (8.14%)	
Anteroseptal	14 (3.35%)	1 (1.16%)	
Inferior	95 (22.73%)	19 (22.09%)	
Lateral	14 (3.35%)	5 (5.82%)	
Posterior	6 (1.44%)	0 (0.00%)	
Past history, n (%)			
Prior AF			< 0.001
No	390 (93.30%)	69 (80.23%)	
Yes	28 (6.70%)	17 (19.77%)	
Prior CHF			0.287
No	359 (85.89%)	70 (81.40%)	
Yes	59 (14.11%)	16 (18.60%)	
Prior hypertension			0.594
No	191 (45.69%)	42 (48.84%)	
Yes	227 (54.31%)	44 (51.16%)	
Prior stroke			0.651
No	388 (92.82%)	81 (94.19%)	
Yes	30 (7.18%)	5 (5.81%)	
Prior CABG			0.939
No	385 (92.11%)	79 (91.86%)	
Yes	33 (7.89%)	7 (8.14%)	
PCI within 24 h			0.587
No	119 (28.47%)	22 (25.58%)	
Yes	299 (71.53%)	64 (74.42%)	
Thrombolytics within 24 h			0.908
No	328 (78.47%)	67 (77.91%)	
Yes	90 (21.53%)	19 (22.09%)	
Myocardial infarction during past 6 months			0.632
No	410 (98.09%)	85 (98.84%)	
Yes	8 (1.91%)	1 (1.16%)	
Cardiac arrest			–
No	418 (82.90%)	86 (17.00%)	
Yes	0	0	
Shock			–
No	418 (82.90%)	86 (17.00%)	
Yes	0	0	

Results in table: Mean (SD) Median (Q1 − Q3)/n (%)

*BUN*, blood urea nitrogen; *AG*, serum anion gap; *RDW*, red blood cell distribution width; *SBP*, systolic blood pressure; *DBP*, diastolic blood pressure; *RR*, respiratory rate; *AMI*, acute myocardial infarction; *AF*, atrial fibrillation; *CHF*, congestive heart failure; *CABG*, coronary artery bypass grafting; *PCI*, percutaneous coronary intervention; h, hours

Gender, age, cirrhosis, diabetes, AMI-location, lactate, APACHE IV score, AF, CHF, hypertension, procedural coronary intervention, stroke, MIDUR had no missing data. Except for SaO_2_, which has 23% missing data, most of the remaining variables have fewer than 10% missing values.

### XGBoost for initial variable screening

Using XGBoost as a pre-experiment, the variables associated with 28-day mortality were most heavily weighted by lactate and APACHE IV score, with lactate being more important than APACHE IV score (Additional file 1: Figure S1).

### Independent predictors in the development cohort

Univariate analysis of the development group showed that the statistically significant risk factors were age, gender, height, weight, hemoglobin, creatinine, BUN, lactate, PH, APACHE IV score, DBP, RR, and prior AF (Table 1).

As the APACHE IV scoring system includes age, RR, PH, creatinine, and BUN, we omitted these five factors before running the generalized linear model to avoid multicollinearity. Ultimately, the following 4 indicators were screened as independent predictors of AMI 28-day mortality in the multivariate logistic regression models: gender, the first sample of admission lactate, APACHE IV, and prior AF (Table 2). We ran a multicollinearity check (VIF step screening methods) based on these four variables before constructing the predicted nomogram, which showed no multicollinearity (Additional file 1: Table S2).

**Table 2:** Multivariate logistic regression model and the Odds ratio of predictors

Variable	*β*	OR (95% CI)	*P-*value
Gender	1.0355		0.0002
Male		1.0	
Female		2.82 (1.62, 4.89)	
Lactate (mmol/L)	0.3848	1.47 (1.29, 1.67)	< 0.0001
APACHE IV score	0.0316	1.03 (1.02, 1.04)	< 0.0001
Prior AF	1.1213		0.0033
No		1.0	
Yes		3.07 (1.45, 6.49)	

*Logistic regression model*: − 5.35472 + 1.03550*(GENDER = Female) + 0.38478*LACTATE + 0.03163*APACHESCORE + 1.12128*(AF = Yes)

*AF*, atrial fibrillation

### Assessment and validation of the nomogram for 28-day mortality

Lactate and APACHE IV score, which were discussed in the background section, were among the four factors that were eventually screened out using rigorous statistical approaches. We initially built and validated a prediction model utilizing these two indicators to compare the improvement of the first sample of admission lactate on the APACHE IV score.

**Fig. 2:**
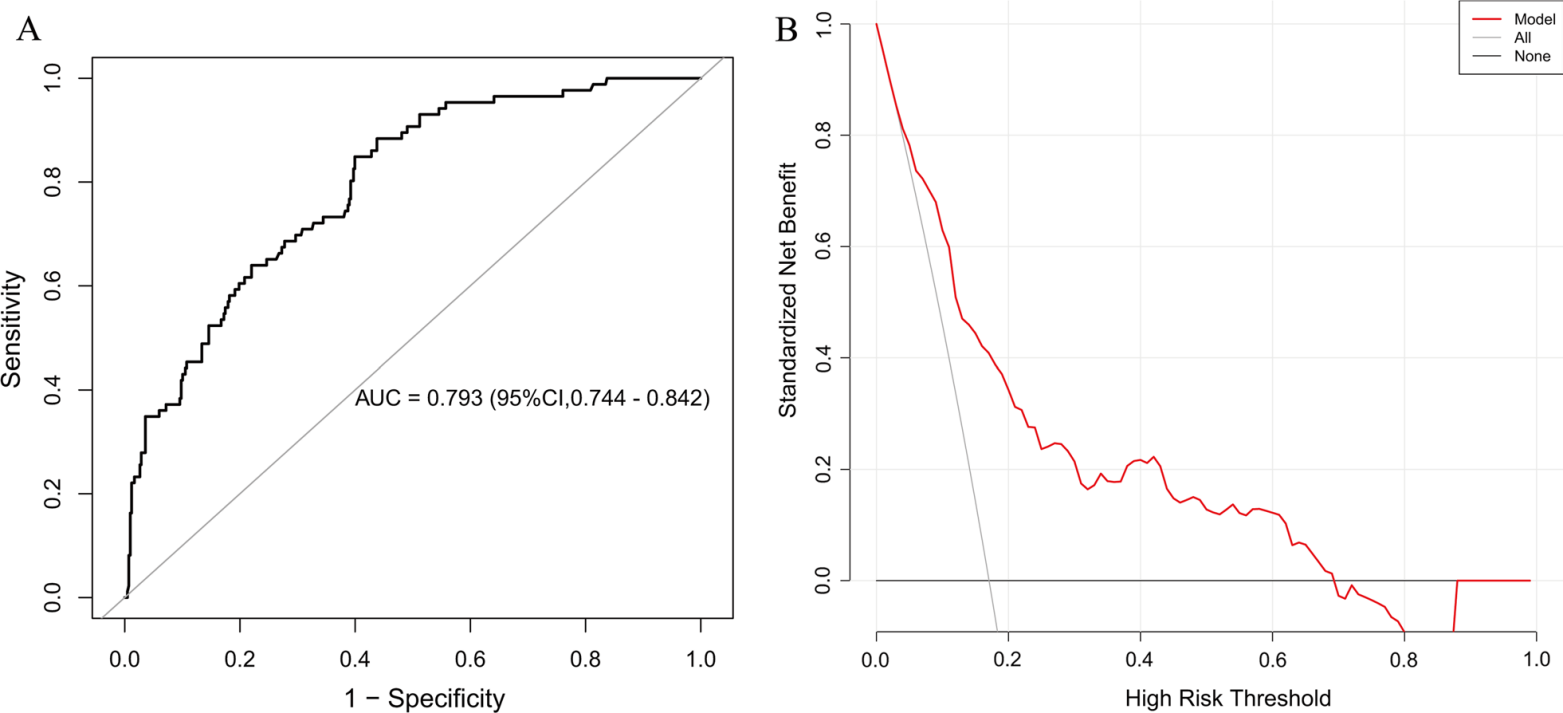
**A** The receiver operating characteristic curve (ROC) and **B** decision curve analysis (DCA) of the first sample of admission lactate combined with APACHE IV score with 28-day mortality. The red solid line represents the predicted estimates

One by one, the Receiver operating characteristic curve (ROC) and DCA curves of lactate and APACHE with 28-day mortality were examined. The AUC for the first sample of admission lactate was 0.713 (95%CI 0.650–0.776) and for the APACHE IV score was 0.733 (95%CI 0.677–0.790). When the threshold probability is between 20 and 65%, the DCA curve for the first sample of admission lactate is higher than the APACHE IV score, as can be seen from the DCA curve (Additional file 1: Figures S2 and S3). When these two variables were modeled simultaneously, the AUC was 0.793 (95%CI 0.744–0.842) and the DCA curve performed better than either variable alone (Fig. 2). The continuous NRI and IDI values were then determined for a model comprising lactate and APACHE IV in comparison to a model containing only APACHE IV. A model containing lactate in addition to APACHE IV had a continuous NRI of 8% (95% CI: − 1.2–17.2%; *p* = 0.0895), driven by correct reclassification of 28-day mortality in 4.7% (*p* = 0.2818) and survival in 3.4% (*p* = 0.0743). Meanwhile, the IDI was 8% (95% CI: − 1.3–17.3%; *p* = 0.0911) (Table 3).

**Table 3:** NRI and IDI analyses for models containing Admission early lactate (A) and Admission early lactate, AF, Gender (B) in addition to APACHE IV score

	NRI	*P*-value	IDI	*P*-value
*(A) Lactate with APACHE IV score*				
Among event subjects	4.7%	0.2818		
Among non-event subjects	3.4%	0.0743		
Overall (95% bootstrap CI)	8% (− 1.2–17.2%)	0.0895	8% (− 1.3–17.3%)	0.0911
*(B) Three variables with APACHE IV score*				
Among event subjects	3.5%	0.4655		
Among non-event subjects	12.9%	< 0.0001		
Overall (95% bootstrap CI)	16.4% (6.1–26.8%)	0.0019	16.4% (6.0–26.8%)	0.0020

*NRI*, Net reclassification improvement; *IDI*, Integrated discrimination improvement; *AF*, atrial fibrillation

**Fig. 3:**
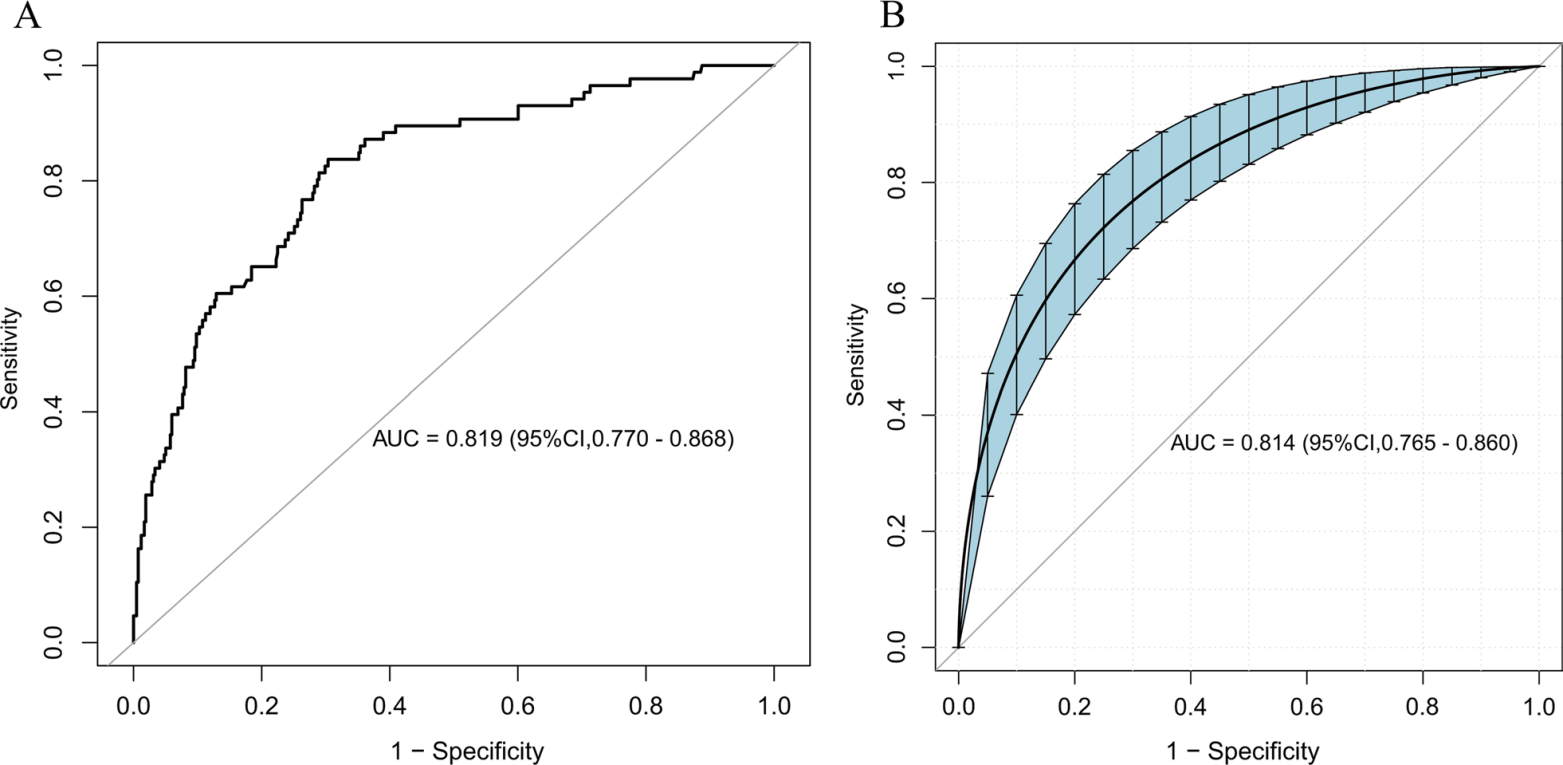
Receiver operating characteristic curve (ROC) of the predictive model and in the internal validation model. The area under the curve (AUC) **A** shows the discrimination ability of the model, and AUC **B** of the internal validation model. The shaded blue portion represents the 95% confidence interval. CI, confidence interval

Further, we took three other variables along with the APACHE IV score, for a total of four variables, also screened in the previous statistical section, for the construction of the prediction model. The prediction model had an AUC of 0.819 (95%CI 0.770–0.868), whereas the internal validation model had an AUC of 0.814 (95%CI 0.765–0.860) (Fig. 3). In further validation group, the AUC for the training group was 0.831(95%CI 0.766–0.896), and the AUC for the validation group was 0.805 (95%CI 0.733–0.878) (Additional file 1: Figure S4).

**Fig. 4:**
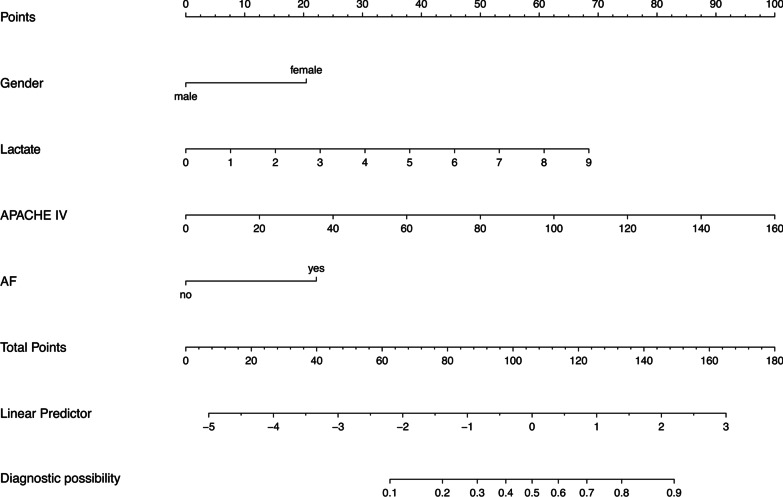
The nomogram scoring system for predicting AMI patients’ 28-day mortality based on APACHE IV, the first sample of admission lactate, gender, and prior atrial fibrillation (AF)

**Fig. 5:**
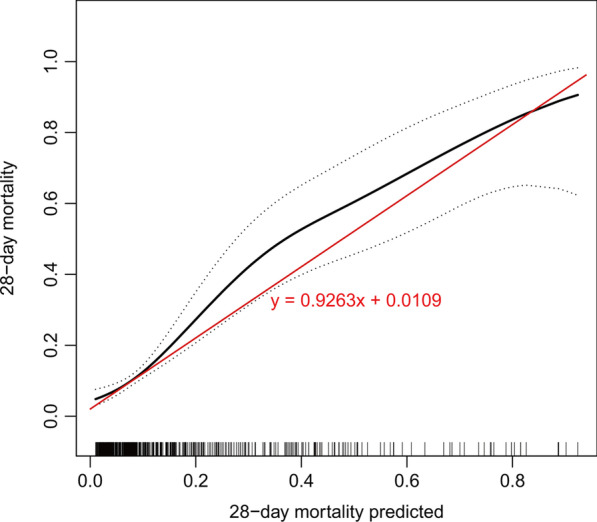
The calibration slope and intercept are almost ideally 1 and 0, respectively (good calibration). It shows a good fit between the predicted risk of 28-day mortality and observed outcomes in patients with acute myocardial infarction (AMI)

**Fig. 6:**
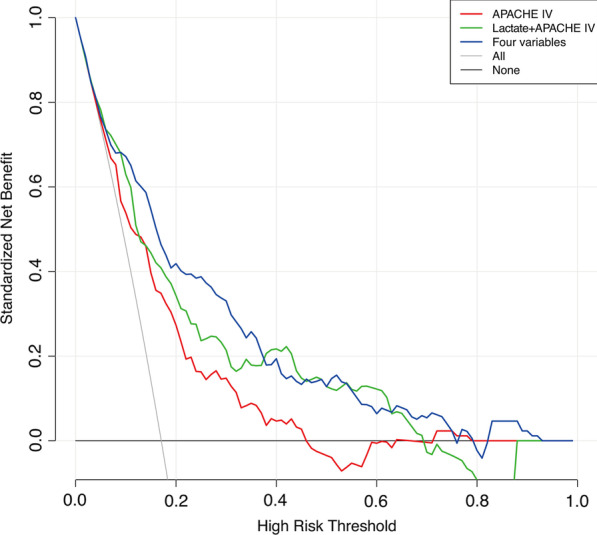
Decision curve analysis (DCA) of three predictive model. The red solid line represents the predicted net benefit of APACHE IV, the green solid line represents the predicted net benefit of lactate joint APACHE IV, and the blue solid line represents the predicted net benefit of the total prediction model for all four variables. Overall, the predictive models for the four variables show better net benefits for the population

Based on the four significant independent predictors, we constructed a nomogram for predicting the hospital 28-day mortality of AMI patients from ICU in the prediction cohort (Fig. 4). The calibration curve of the nomogram for the 28-day mortality prediction model had an intercept of 0.9263 and a slope of 0.0109, which showed good accuracy compared with the actual world (Fig. 5). Additionally, the predictive model composed of these four variables outperforms a single APACHE in terms of NRI and IDI. The NRI was 16.4% (95% CI: 6.1–26.8%; *p* = 0.0019), driven by correct reclassification of 28-day mortality in 3.5% (*p* = 0.4655) and survival in 12.9% (*p* < 0.0001), and the IDI was 16.4% (95% CI: 6.0–26.8%; *p* = 0.0020) (Table 3). As illustrated in Fig. 6, the DCA curves for the four variables prediction model demonstrated significant better net improvements across a range of mortality risks when compared to single APACHE IV or lactate plus APACHE IV.

### Comparison with the M-CARS model

The M-CARS model has 7 variables: the admission Braden skin score, the admission red blood cell distribution width (RDW), the admission BUN, the admission serum anion gap, the admission diagnosis of cardiac arrest, the admission diagnosis of shock, and the admission diagnosis of respiratory failure. These admission diagnoses were not mutually exclusive and the primary admission diagnosis could not be determined. The AUC for the M-CARS model to predict 28-day mortality in AMI patients was 0.672, which was less than the full model we made, which was 0.819 (Additional file 1: Figure S5). Among the 504 AMI patients, the admission diagnosis of both cardiac arrest and shock was zero, so only five variables were used to predict AMI patients (Table 1). For the DCA curves, our full model showed better population benefit at almost all high-risk thresholds (Additional file 1: Figure S6). In addition, our full model had an overall NRI of 23.02% (95% CI: 9.54–36.50%; *p* = 0.0008) and an IDI of 23.02% (95% CI: 9.47–36.56%; *p* = 0.0009) compared with the M-CARS model (Additional file 1: Table S3).

### Sensitivity analysis

To determine the validity of our findings, we conducted various sensitivity analyses. By means of multiple interpolation, we regenerated the missing data into 5 sets and applied them to univariate analysis and generalized linear models. Thus, the results of the five data sets were combined, and their screened variables and effect values were essentially identical to those of the original data sets (Additional file 1: Tables S4 and S5).

### Web calculator of dynamic nomogram

To increase the clinical application of predictive models, web calculator (https://doctorsong.shinyapps.io/AMInomogram/) was developed that can be very easily performed by medical practitioners, thus making the application much less difficult. Concerning the observation window, gender and prior AF (if a patient had AF on the first electrocardiogram but it wasn't known before, it was also counted as prior AF) can be gotten at the time of admission consultation. Lactate can be gotten from the first lactate value on the first day of admission, and the dynamic nomogram can be used to predict 28-day mortality in AMI patients after the APACHE IV score is gotten.

### Exploration of the applicability of the model

From Additional file 1: Tables S6, we can see that the baseline disease status of the 6817 AMI patients in the eICU database with or without lactate measurement was mostly different in the following ways: combined diabetes, prior AF, prior CHF, and prior stroke. Therefore, the dynamic nomogram we developed should be more suitable for AMI patients with combined diabetes, prior CHF, prior AF, and prior stroke.

## Discussion

Our study demonstrated that the APACHE IV score, in combination with several other variables proven in previous studies, improved the clinical predictive power of the APACHE IV model for AMI patients. The strengths of this study are the multicenter collection and the vast number of patients included. Our nomogram model incorporates risk factors within the early 24 h of admission, thus enabling assessment of the prognosis of AMI patients in the earliest 24 h. Most of the predictive information was contained in the four most relevant factors: the first sample of admission lactate, gender, prior AF, and APACHE IV, which were put into the nomogram. Compared to the single APACHE IV model, the combination of the other three variables increased the predictive power of the overall model by 16.4%. In addition, the model was better validated, and its clinical calibration showed better results. Eventually, a web nomogram was developed to ease the burden in clinical applications. Nomograms are clinical evidence-based tools used to guide clinical decision-making and provide accurate and repeatable predictions that do not require computer software to interpret [32]. Accurate identification and categorisation of AMI patients by nomogram facilitates, on the one hand, the identification of high-risk patients to benefit from pharmacological and interventional treatment as early as possible and, on the other hand, facilitates better explanation of the condition to patients and families, informing them of the prognosis and improving the doctor-patient relationship.

In the introduction, this study has preliminarily addressed some of the issues regarding TIMI and GRACE scores, and although they are currently the main prognostic scoring tools for AMI patients, their limitations cannot be ignored. On the one hand, the TIMI risk score is mainly used for ST-segment elevation myocardial infarction (STEMI) and lacks systemic physiological parameters, which have been shown to be inefficient in non-STEMI (NSTEMI), while on the other hand, the GRACE score fails to cover coronary risk factors, the patient's previous cardiovascular history, and other indicators that may have an impact on the prognosis of AMI patients. APACHE IV is a system for evaluating the whole-body physiology, including key physiological parameters and some past medical history, and can compensate for the limitations of TIMI and GRACE in patients with AMI. Internationally, reports on the application of the APACHE IV are relatively scarce, especially in patients with AMI, suggesting that its application has yet to be promoted. The APACHE IV scoring system is an emerging critical care scoring system that evaluates the degree of criticality and can predict prognosis. However, the APACHE IV scoring system was designed to be applied in integrated ICUs, and therefore, it is essential to explore whether it can better evaluate the degree of criticality of cardiovascular disease. Zimmerman JE [8] concluded that the APACHE IV prediction of in-hospital mortality is meaningful and should be implemented in ICUs. However, the accuracy of the prediction should be dynamic and tested multiple times over time, and should be revised and updated when the accuracy decreases. Antonio Paulo Nassar Jr [10] found that the calibration of the APACHE IV was poor in a study that included 1229 patients admitted with acute coronary syndrome (ACS). The accuracy of the APACHE IV in predicting AMI patients should be improved by adding or subtracting variables according to the characteristics of the cardiovascular disease.

Lactate, as a readily and rapidly evaluated metabolite, has been under long-term study in patients with acute heart disease and can help clinical practitioners assess patient prognosis [16]. Higher lactate levels in STEMI patients are associated with 30-day mortality, especially lactate ≥ 1.8 mmol/L [39]. In a study including 766 people, patients were ACS patients who underwent coronary artery bypass grafting and lactate was found to be associated with 30-day mortality [40]. Additionally, increased lactate levels (≥ 1.8 mmol/L) at admission were an independent predictor of 30-day and 180-day all-cause death in a trial of 1865 patients with ACS [41]. Stavros G. Drakos' team study identifies the pyruvate-lactate axis as a key node in the homeostasis of cardiac function. This study found that alterations in the pyruvate-lactate axis are an early feature of cardiac hypertrophy and failure, and that myocardial cell failure is a strong predictor of death [20]. Baysan et al. showed that in patients with sepsis, lactate within 24 h of admission increased the predictive efficacy of APACHE IV [19]. These studies above suggest that early lactate elevation may play a key predictive role in long-term mortality. However, to our knowledge, there are no risk prediction tools for AMI in combination with early lactate to date. Therefore, there is an urgent need for a well-performing AMI prediction model that includes lactate early in hospital admission.

Yves Cottin et al. found that atrial fibrillation is a common complication associated with increased mortality in patients with all types of AMI. In the context of risk profiles for non-obstructive coronary myocardial infarction, atrial fibrillation is associated with frequent coronary embolism, which is often easily underestimated [42]. Prashanthan Sanders et al. revealed that multivariate analysis showed that atrial fibrillation independently predicted myocardial infarction [HR, 2.41 (1.74, 3.35), *p* < 0.001] and further studies are necessary to determine the pathogenesis of myocardial infarction in the setting of atrial fibrillation [43]. Alvaro Alonso et al. observed a gender difference between AF and AMI, with women having a greater risk of MI in AF compared to men [44]. In summary, previous history of atrial fibrillation and gender play an important role in the prognosis of AMI patients, which is consistent with our findings from multiple regression analysis. Possible myocardial fibrosis due to a previous history of atrial fibrillation may lead to heart failure in AMI patients, which in turn may worsen the condition as well as affect the prognosis. Regarding the relationship between gender and AMI, particularly in women, some of the following reasons may explain the difference in prognosis of AMI by gender differences: 1. Some female patients are in the perimenopausal period and symptoms of menopause interfere with the perception of chest pain. 2. A higher proportion of female patients have combined diabetes mellitus and peripheral neuropathy in diabetes, which raises the pain threshold. 3. Premenopausal women with coronary artery disease often have typical chest pain, whereas patients of advanced age often have no typical clinical symptoms.

Predictive models are evaluated in three main areas, namely discrimination, calibration, and clinical validity. The discrimination is mainly represented by the AUC, NRI and IDI. Calibration is represented by the calibration plot. Clinical validity is represented by the DCA curve. In this study, the APACHE IV score was combined with early admission lactate, previous AF, and gender to construct the final model. The prediction model had an AUC of 0.819 (95%CI 0.770–0.868), whereas the internal validation model had an AUC of 0.814 (95%CI 0.765–0.860). The predictive model composed of these four variables outperformed a single APACHE IV in terms of NRI and IDI. The NRI was 16.4% (95% CI: 6.1–26.8%; *p* = 0.0019), driven by correct reclassification of 28-day mortality in 3.5% (*p* = 0.4655) and survival in 12.9% (*p* < 0.0001), and the IDI was 16.4% (95% CI: 6.0–26.8%; *p* = 0.0020). When APACHE IV was modeled in conjunction with lactate alone, the model had a continuous NRI of 8% (95% CI: − 1.2–17.2%; *p* = 0.0895), driven by correct reclassification of 28-day mortality in 4.7% (*p* = 0.2818) and survival in 3.4% (*p* = 0.0743). Lactate accounted for nearly half of the total NRI, which showed that lactate was the most important of the other three variables. This showed that the prediction model constructed by APACHE IV in conjunction with the other three variables had a good degree of discrimination. Immediately following the analysis of the calibration plot, the slope was close to 1 and the intercept was close to 0. The predicted mortality line was close to the actual value curve, which meant that the overall prediction model was reflective of real-world conditions. Finally, analysis of the DCA curves showed that the overall prediction model had a much better curve than the single APACHE IV, which overall indicated that the population benefited more from the prediction model in this study than from the APACHE IV model alone.

After constructing and validating our model, we compared it with the M-CARS model. The results showed that our full model outperformed the M-CARS model in terms of AUC, DCA curve, NRI, and IDI. As mentioned in the introduction, the M-CARS model was constructed using patient data from the entire CICU, and the predictive efficacy for AMI patients may not be accurate. This hypothesis was also verified after our test on AMI patients. Reviewing the M-CARS study, we found that the proportion of patients admitted with a diagnosis of cardiac arrest and shock in that study was 12.0% and 13.6% of the 10,004 patients, respectively [15]. However, none of our AMI patients had a comorbid diagnosis of cardiac arrest or shock at the time of admission. Even among the 6817 AMI patients in the eICU database, only one was admitted with a comorbid cardiac arrest diagnosis, and none were diagnosed with shock. This would explain why the predictive efficacy of the M-CARS model decreases in AMI patients.

The acquisition of the APACHE IV score requires a certain amount of time for clinical decision-making. However, lactate, prior AF, and gender are very easy to obtain in the early stage of admission. To improve the efficiency of the clinical application of this study model, a dynamic nomogram was created for the website app, which allows clinicians to easily obtain specific predictive values in less than 1 min. In less than 1 min, the prediction accuracy of AMI patients improved by 16.4%.

In recent years, due to the rapid development of big data technology and parallel computing, the massive amount of data and the high speed of computational efficiency have led to the widespread interest in machine learning in the medical field. XGBoost is a new and effective machine learning method that has come out in the past few years. In this study, we tried to use XGBoost to help us screen variables at the start. In recent years, there have been many articles exploring the role of machine learning in predicting mortality in AMI patients. Although these studies were not early admission predictions of mortality in AMI, various forms of machine learning methods were used to predict mortality in AMI. In 2019, Kwon et al. constructed models for deep machine learning on hospitalization and 12-month mortality in AMI patients and showed superiority over GRACE and TIMI scores [45]. In 2021, Lee et al. constructed machine learning methods by logistic regression with regularization, random forest, support vector machine, and extreme gradient boosting to construct 3-month, 12-month, and in-hospital mortality prediction models for AMI patients. The results showed that machine learning outperformed traditional prediction models [46]. In 2022, Xiao et al. employed six machine learning methods to construct predictive models for the occurrence of major adverse cardiovascular events (MACEs) in AMI patients. The results showed that the best performer was the random forest model, with an AUC of (0.749, 0.644–0.853) [47]. However, these models did not explore the calibration of the models and the DCA curves, which are very important indicators of the model efficacy. In 2022, Khera et al.'s machine learning calibrated the model, but they did not explore the DCA curve either [48]. In fact, another very big problem with machine learning, as described above, is that it is not directly usable by clinical workers. In other words, there are very significant limitations to clinical help. This problem is also common to many other machine learning applications. However, our study explored not only the calibration degree and DCA curves of the prediction model but, most importantly, constructed an online dynamic nomogram that can be used directly. As described above, clinicians can improve the predictive efficacy of APACHE IV scores by 16.4% in less than 1 min.

### Limitations

Our study has limitations. First, the model constructed in this study was developed primarily for a predominantly white population, and the model's population was derived from tertiary referral centers [49]; therefore, its validity in other populations as well as in the community remains to be tested. In addition, TIMI and GRACE scores were not available in the eICU database, and unfortunately, we were unable to quantitatively compare our model with these two models. What’s more, as with all observational studies, not all variables could be collected in this study, and some data were missing. For AMI patients, some variables such as left ventricular ejection fraction, mechanical complications, short-term circulatory support, Killip classification, and vasoactive drugs were completely missing or very severely missing, making it impossible to apply them to our statistical analysis, which is a very significant limitation of this study. Finally, for lactate, nearly 90% of AMI patients have missing lactate data. Therefore, our prediction model may not be applicable to other AMI populations, and the model should not be extrapolated to other AMI populations. The applicability of the model to other AMI patients requires further validation. The dynamic nomogram we developed should be more suitable for AMI patients with combined diabetes, prior CHF, prior AF, and prior stroke.

## Conclusion

In conclusion, the prediction model constructed by APACHE IV in combination with the first sample of admission lactate, prior AF, and gender outperformed the APACHE IV scoring system alone in predicting 28-day mortality in AMI. Of these three variables combined, the best performer was the first sample of admission lactate, confirming the importance of early lactate for disease prognosis in AMI patients. The prediction model was published via a website app, allowing clinicians to improve the predictive efficacy of the APACHE IV score by 16.4% in less than 1 min. In addition, the dynamic nomogram we developed should be more suitable for AMI patients with combined diabetes, prior CHF, prior AF, and prior stroke.

## Supplementary Information

**Additional file 1**: **Table S1**. Comparison of non-Q AMI and anterior AMI population characteristics. **Table S2**. Multicollinearity check. **Table S3**. NRI and IDI analysis of the improvement of our full model on the M-CARS model. **Table S4**. Multiple interpolation for the univariate analysis. **Table S5**. Multiple interpolation for the generalized linear models. **Table S6**. The applicability of the model was explored by comparing the baseline disease of patients admitted with or without lactate measurement in the eICU database of 6817 AMI patients. **Fig. S1**. Using XGBoost as a pre-experiment, the variables associated with 28-day mortality were screened. **Fig. S2**. One by one, the ROC curves of lactate and APACHE IV with 28-day mortality were examined. **Fig. S3**. One by one, the DCA curves of lactate and APACHE IV with 28-day mortality were examined. **Fig. S4**. The AUC for the training group was 0.831, and the AUC for the validation group was 0.805. **Fig. S5**. The AUC for the M-CARS model was 0.672, and the AUC for our full model was 0.819. **Fig. S6**. The DCA curves of M-CARS model and out full model with 28-day mortality were examined.

## Data Availability

The datasets generated and/or analysed during the current study are available in the eICU Collaborative Research Database, which can be accessed on https://physionet.org/content/eicu-crd/2.0/

## Abbreviations

APACHE: Acute physiology and chronic health evaluation
AUC: Area under the receiver operating characteristic curve
CI: Confidence interval
AMI: Acute myocardial infarction
NRI: Net reclassification improvement
IDI: Integrated discrimination improvement
DCA: Decision curve analysis
STEMI: ST-elevation myocardial infarction
NSTEMI: Non-STEMI
TRIPOD: Transparent reporting of a multivariable prediction model for individual prognosis or diagnosis
BUN: Blood urea nitrogen
SBP: Systolic blood pressure
DBP: Diastolic blood pressure
HR: Heart rate
RR: Respiratory rate
SaO_2_: Percutaneous oxygen saturation
AF: Atrial fibrillation
MIDUR: Myocardial infarction in the past 6 months
CHF: Congestive heart failure
ROC: Receiver operating characteristic
CABG: Coronary artery bypass grafting
PCI: Percutaneous coronary intervention
