# Supplementary material for: A simple APACHE IV risk dynamic nomogram that incorporates early admitted lactate for the initial assessment of 28-day mortality in critically ill patients with acute myocardial infarction
Source: BMC Cardiovasc Disord. 2022 Nov 24;22:502. doi: 10.1186/s12872-022-02960-8 (PMC9700900; doi:10.1186/s12872-022-02960-8)
Supplement: Supplementary file 1 — Additional file 1: Table S1. Comparison of non-Q AMI and anterior AMI population characteristics. Table S2. Multicollinearity check. Table S3. NRI and IDI analysis of the improvement of our full model on the M-CARS model. Table S4. Multiple interpolation for the univariate analysis. Table S5. Multiple interpolation for the generalized linear models. Table S6. The applicability of the model was explored by comparing the baseline disease of patients admitted with or without lactate measurement in the eICU database of 6817 AMI patients. Fig. S1. Using XGBoost as a pre-experiment, the variables associated with 28-day mortality were screened. Fig. S2. One by one, the ROC curves of lactate and APACHE IV with 28-day mortality were examined. Fig. S3. One by one, the DCA curves of lactate and APACHE IV with 28-day mortality were examined. Fig. S4. The AUC for the training group was 0.831, and the AUC for the validation group was 0.805. Fig. S5. The AUC for the M-CARS model was 0.672, and the AUC for our full model was 0.819. Fig. S6. The DCA curves of M-CARS model and out full model with 28-day mortality were examined. [file 12872_2022_2960_MOESM1_ESM.docx]

**Additional file 1:** Supplemental Appendices.

**Additional file 1: Table S1.** Comparison of non-Q AMI and anterior AMI population characteristics

**Additional file 1: Table S2.** Multicollinearity check.

**Additional file 1: Table S3.** NRI and IDI analysis of the improvement of our full model on the M-CARS model

**Additional file 1: Table S4.** Multiple interpolation for the univariate analysis

**Additional file 1: Table S5.** Multiple interpolation for the generalized linear models

**Additional file 1: Table S6.** The applicability of the model was explored by comparing the baseline disease of patients admitted with or without lactate measurement in the eICU database of 6817 AMI patients.

**Additional file 1: Figure S1.** Using XGBoost as a pre-experiment, the variables associated with 28-day mortality were screened.

**Additional file 1: Figure S2.** One by one, the ROC curves of lactate and APACHE IV with 28-day mortality were examined

**Additional file 1: Figure S3.** One by one, the DCA curves of lactate and APACHE IV with 28-day mortality were examined

**Additional file 1: Figure S4.** The AUC for the training group was 0.831, and the AUC for the validation group was 0.805

**Additional file 1: Figure S5.** The AUC for the M-CARS model was 0.672, and the AUC for our full model was 0.819.

**Additional file 1: Figure S6.** The DCA curves of M-CARS model and out full model with 28-day mortality were examined.

**Additional file 1: Table S1. Comparison of non-Q AMI and anterior AMI population characteristics**

| AMI-location | Non-Q | Anterior | P-value |
| --- | --- | --- | --- |
| No, n (%) | 224 (71.3%) | 90 (28.7%) |  |
| 28-day mortality |  |  | 0.874 |
| No | 185 (82.59%) | 75 (83.33%) |  |
| Yes | 39 (17.41%) | 15 (16.67%) |  |
| Age | 70.96±11.23 | 65.20±14.64 | <0.001 |
| Prior AF |  |  | 0.044 |
| No | 201 (89.73%) | 87 (96.67%) |  |
| Yes | 23 (10.27%) | 3 (3.33%) |  |
| Prior CHF |  |  | <0.001 |
| No | 177 (79.02%) | 86 (95.56%) |  |
| Yes | 47 (20.98%) | 4 (4.44%) |  |
| Thrombolytics within 24h |  |  | 0.023 |
| No | 191 (85.27%) | 67 (74.44%) |  |
| Yes | 33 (14.73%) | 23 (25.56%) |  |
| PCI within 24h |  |  | 0.005 |
| No | 84 (37.50%) | 19 (21.11%) |  |
| Yes | 140 (62.50%) | 71 (78.89%) |  |
| Prior CABG |  |  | 0.370 |
| No | 199 (88.84%) | 83 (92.22%) |  |
| Yes | 25 (11.16%) | 7 (7.78%) |  |

Results in table: Mean±SD/ n (%)

**Additional file 1: Table S2. Multicollinearity check.**

|  | VIF |
| --- | --- |
| GENDER | 1 |
| LACTATE | 1.1 |
| APACHESCORE | 1.1 |
| AF | 1 |

Variables removed: None
Variables selected: GENDER LACTATE APACHESCORE AF

**Additional file 1: Table S3. NRI and IDI analysis of the improvement of our full model on the M-CARS model**

|  |  | NRI | *P*-value | IDI | *P*-value |
| --- | --- | --- | --- | --- | --- |
| Our full model vs. M-CARS | Among event subjects | 23.26% | 0.0002 |  |  |
|  | Among non-event subjects | -0.24% | 0.9309 |  |  |
|  | Overall (95% bootstrap CI) | 23.02% (9.54-36.50%) | 0.0008 | 23.02% (9.47-36.56%) | 0.0009 |

**Additional file 1: Table S4. Multiple interpolation for the univariate analysis**

| variables | Pro-imputation 1 | Pro-imputation 2 | Pro-imputation 3 | Pro-imputation 4 | Pro-imputation 5 | **Combined OR** | *P*-value |
| --- | --- | --- | --- | --- | --- | --- | --- |
| Height | 0.03(0.00,0.25) | 0.04(0.00,0.36) | 0.03(0.00,0.31) | 0.04(0.00,0.35) | 0.04(0.00,0.38) | 0.04(0.00,0.36) | 0.0025 |
| Weight | 0.98(0.97,0.99) | 0.98(0.97,0.99) | 0.98(0.97,0.99) | 0.98(0.97,0.99) | 0.98(0.97,0.99) | 0.98(0.97,0.99) | 0.0001 |
| Hgb | 0.90(0.81,1.00) | 0.90(0.81,1.00) | 0.89(0.80,1.00) | 0.90(0.81,1.00) | 0.89(0.80,0.99) | 0.90(0.80,1.00) | 0.0453 |
| Glucose | 1.05(1.00,1.10) | 1.05(1.00,1.10) | 1.05(1.00,1.10) | 1.05(1.01,1.10) | 1.05(1.00,1.10) | 1.05(1.00,1.10) | 0.0406 |
| Creatinine | 1.56(1.25,1.96) | 1.56(1.24,1.96) | 1.56(1.24,1.96) | 1.55(1.24,1.95) | 1.56(1.24,1.95) | 1.56(1.24,1.96) | 0.0001 |
| BUN | 1.03(1.01,1.04) | 1.03(1.01,1.04) | 1.02(1.01,1.04) | 1.02(1.01,1.04) | 1.02(1.01,1.04) | 1.02(1.01,1.04) | 0.0098 |
| SBP | 0.99(0.98,1.00) | 0.99(0.98,1.00) | 0.99(0.98,1.00) | 0.99(0.98,1.00) | 0.99(0.98,1.00) | 0.99(0.98,1.00) | 0.0511 |
| DBP | 0.98(0.96,0.99) | 0.98(0.97,1.00) | 0.98(0.96,0.99) | 0.98(0.97,1.00) | 0.98(0.97,1.00) | 0.98(0.97,1.00) | 0.0096 |
| HR | 1.00(0.99,1.01) | 1.00(0.99,1.01) | 1.00(0.99,1.01) | 1.00(0.99,1.01) | 1.00(0.99,1.01) | 1.00(0.99,1.01) | 1.0000 |
| RR | 1.06(1.02,1.10) | 1.06(1.02,1.11) | 1.06(1.02,1.11) | 1.06(1.02,1.11) | 1.07(1.03,1.11) | 1.06(1.02,1.11) | 0.0043 |
| SaO2 | 1.04(0.98,1.11) | 1.07(1.00,1.14) | 1.03(0.97,1.09) | 1.05(0.98,1.11) | 1.04(0.98,1.11) | 1.05(0.98,1.12) | 0.1977 |

**Additional file 1: Table S5. Multiple interpolation for the** **generalized linear models**

| variables | Pro-imputation 1 | Pro-imputation 2 | Pro-imputation 3 | Pro-imputation 4 | Pro-imputation 5 | **Combined OR** | *P*-value |
| --- | --- | --- | --- | --- | --- | --- | --- |
| lactate | 1.475(1.297,1.679) | 1.469(1.291,1.672) | 1.469(1.291,1.672) | 1.469(1.291,1.672) | 1.469(1.291,1.672) | 1.470(1.292,1.673) 0.0000 | 0.0000 |
| apache | 1.032(1.020,1.044) | 1.032(1.020,1.044) | 1.032(1.020,1.044) | 1.032(1.020,1.044) | 1.032(1.020,1.044) | 1.032(1.020,1.044) 0.0000 | 0.0000 |
| AF | 3.159(1.496,6.669) | 3.069(1.452,6.487) | 3.069(1.452,6.487) | 3.069(1.452,6.487) | 3.069(1.452,6.487) | 3.087(1.460,6.526) 0.0032 | 0.0032 |
| gender | 1.845(0.841,4.050) | 2.817(1.623,4.888) | 2.817(1.623,4.888) | 2.817(1.623,4.888) | 2.817(1.623,4.888) | 2.588(1.272,5.265) 0.0087 | 0.0087 |

| Additional file 1: Table S6. The applicability of the model was explored by comparing the baseline disease of patients admitted with or without lactate measurement in the eICU database of 6817 AMI patients. | | | |
| --- | --- | --- | --- |
| Variable | **Whether lactate was measured** | | ***P-*value** |
|  | **No (n = 6145)** | **Yes (n = 672)** |  |
| Age (years) | 64.36 (13.30) | 68.91 (12.67) | <0.001 |
| APACHE IV score | 40.61 (17.02) | 61.56 (25.74) | <0.001 |
| Glucose (mmol/L) | 6.56 (5.56-8.33) | 8.22 (6.50-11.78) | <0.001 |
| Diabetes |  |  | **<0.001** |
| No | 4778 (77.75%) | 471 (70.09%) |  |
| Yes | 1367 (22.25%) | 201 (29.91%) |  |
| Prior AF |  |  | **<0.001** |
| No | 5831 (94.89%) | 615 (91.52%) |  |
| Yes | 314 (5.11%) | 57 (8.48%) |  |
| Prior CHF |  |  | **<0.001** |
| No | 5577 (90.76%) | 559 (83.18%) |  |
| Yes | 568 (9.24%) | 113 (16.82%) |  |
| Prior Hypertension |  |  | 0.538 |
| No | 3261 (53.07%) | 365 (54.32%) |  |
| Yes | 2884 (46.93%) | 307 (45.68%) |  |
| Prior Stroke |  |  | **0.041** |
| No | 5822 (94.74%) | 624 (92.86%) |  |
| Yes | 323 (5.26%) | 48 (7.14%) |  |
| MI during past 6 months |  |  | 0.486 |
| No | 5970 (97.15%) | 656 (97.62%) |  |
| Yes | 175 (2.85%) | 16 (2.38%) |  |
| Prior CABG |  |  | 0.666 |
| No | 5640 (91.78%) | 620 (92.26%) |  |
| Yes | 505 (8.22%) | 52 (7.74%) |  |

Results in table: Mean (SD) Median (Q1-Q3) / n (%)


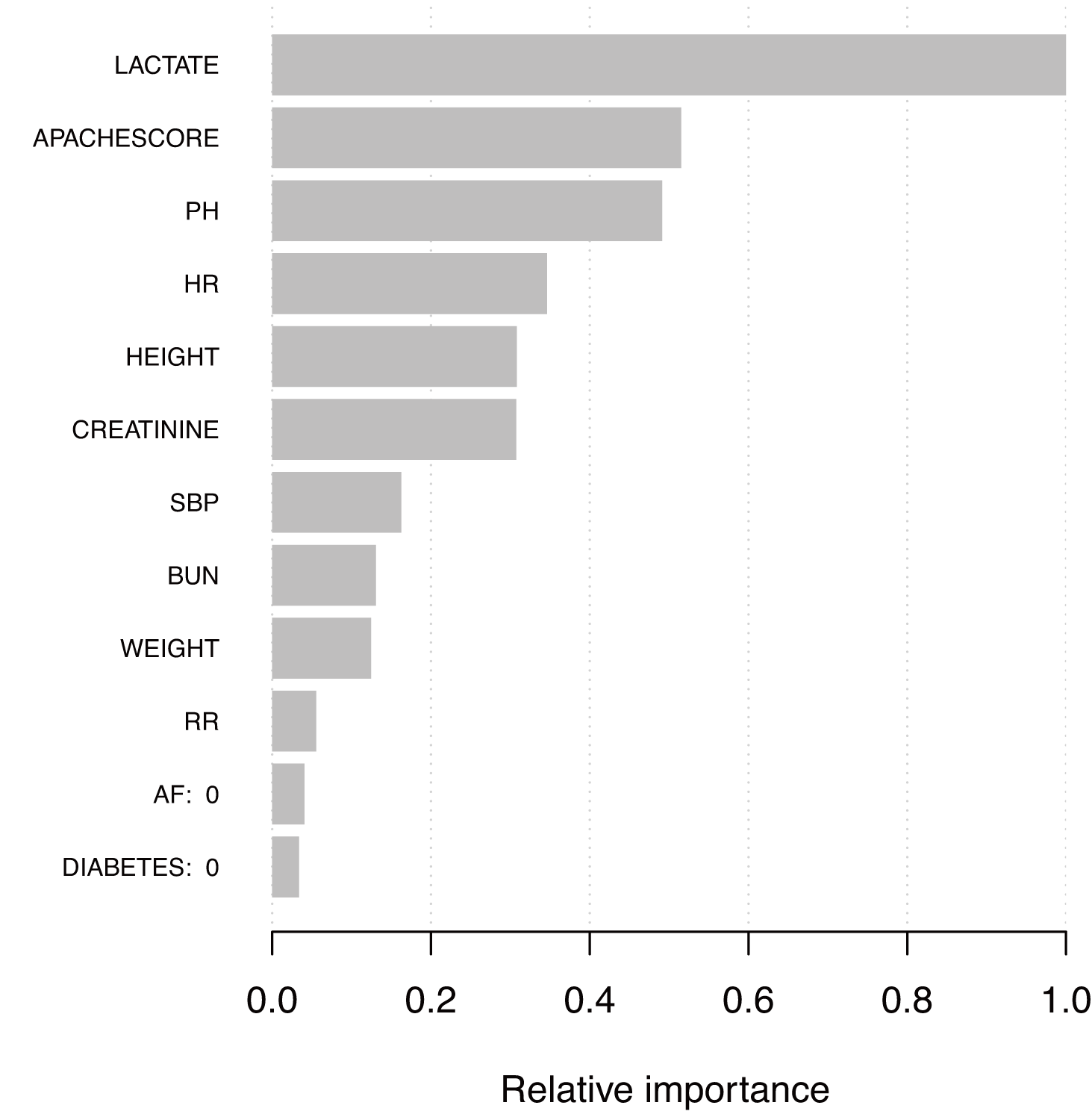


**Additional file 1: Figure S1.** Using XGBoost as a pre-experiment, the variables associated with 28-day mortality were screened.


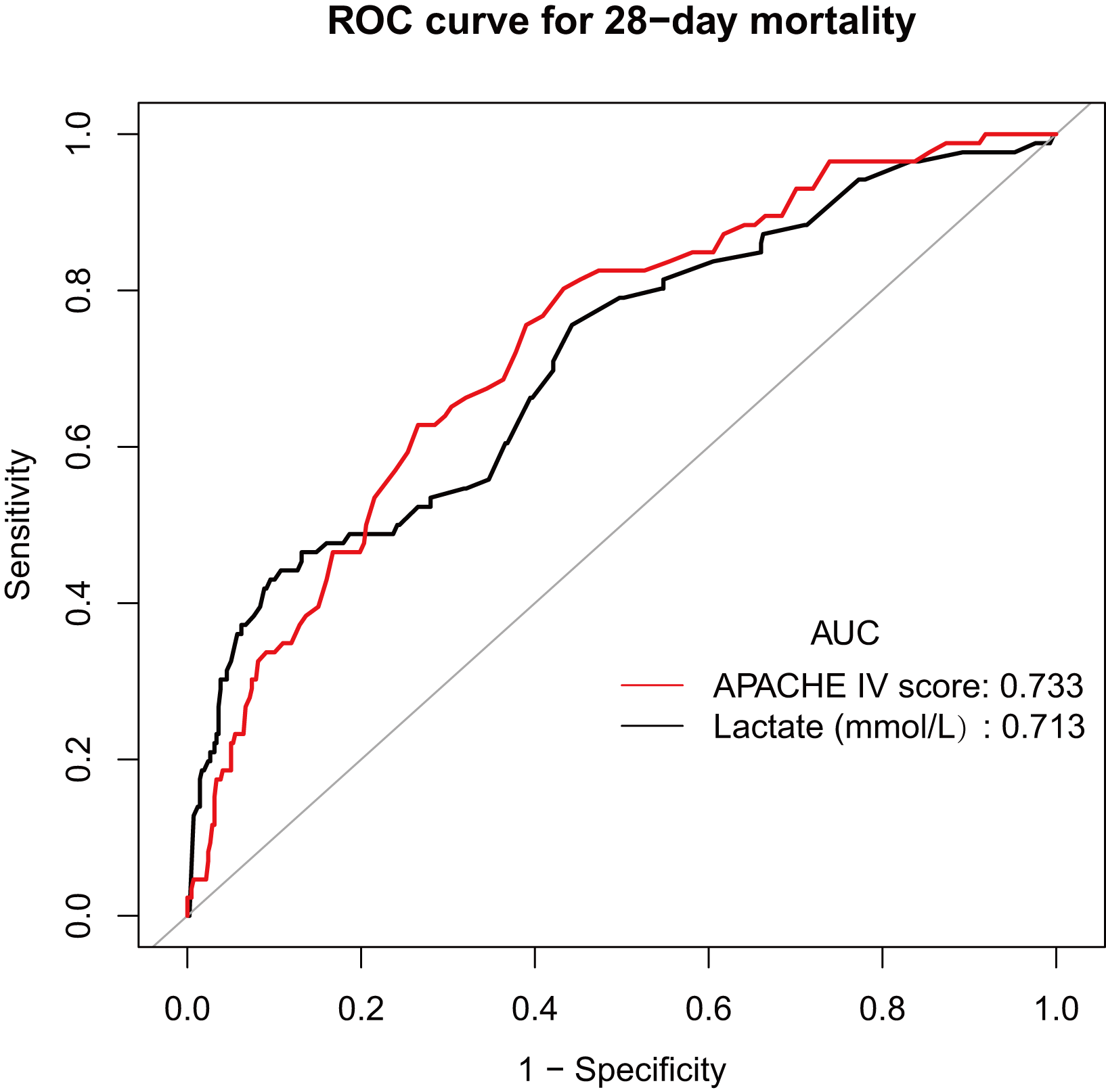


**Additional file 1: Figure S2.** One by one, the ROC curves of lactate and APACHE IV with 28-day mortality were examined


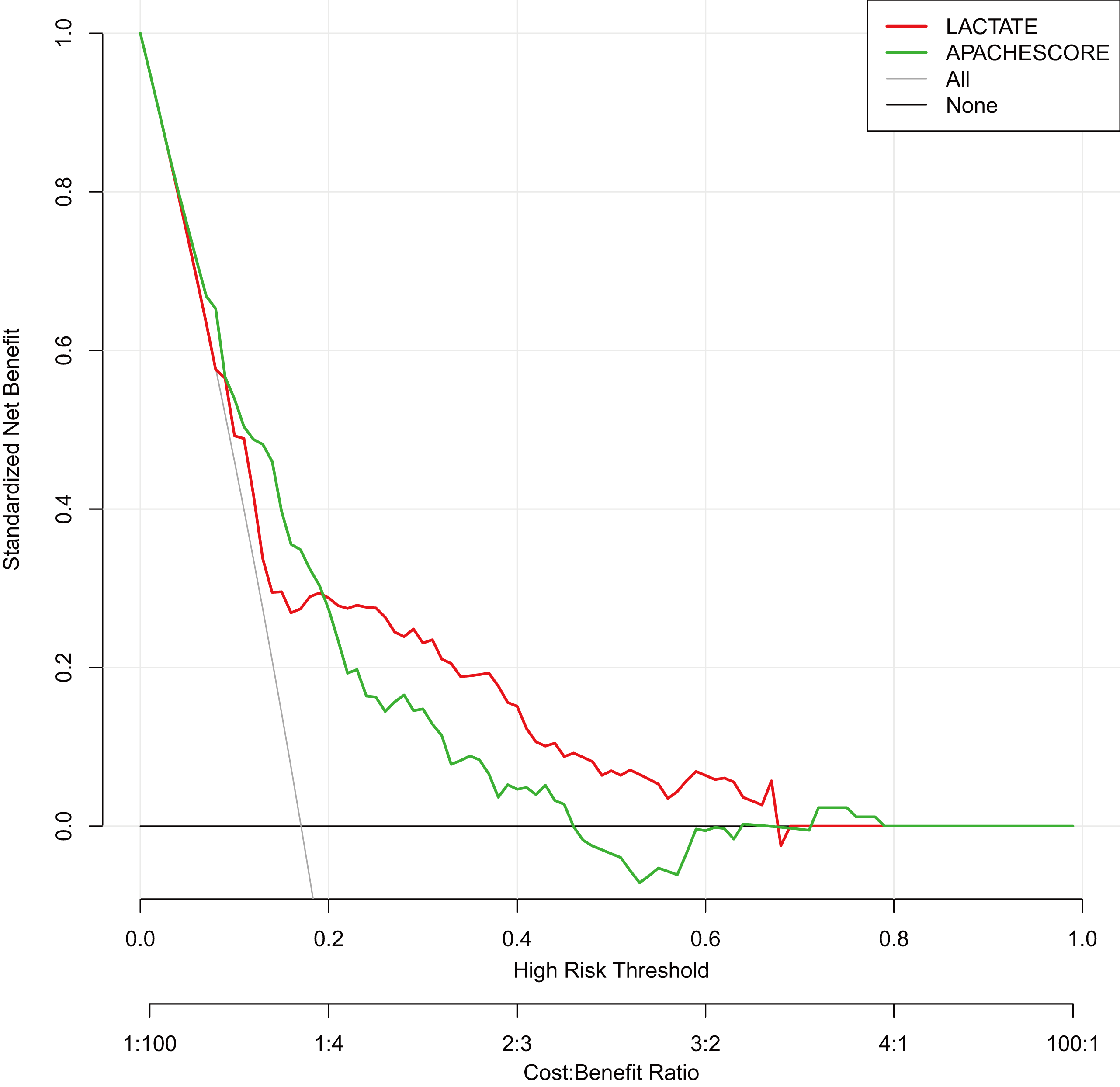


**Additional file 1: Figure S3.** One by one, the DCA curves of lactate and APACHE IV with 28-day mortality were examined.


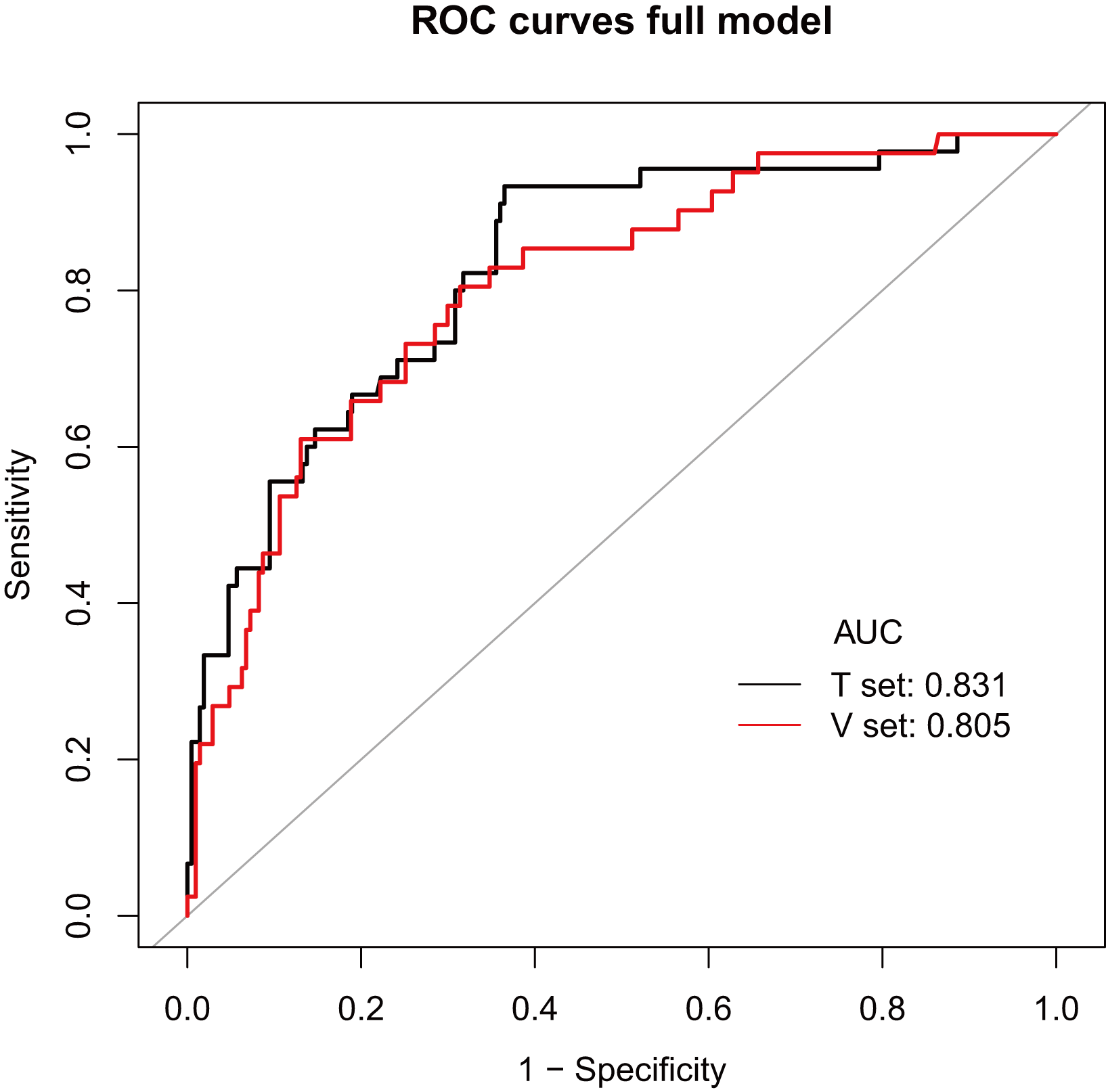


**Additional file 1: Figure S4.** The AUC for the training group was 0.831, and the AUC for the validation group was 0.805


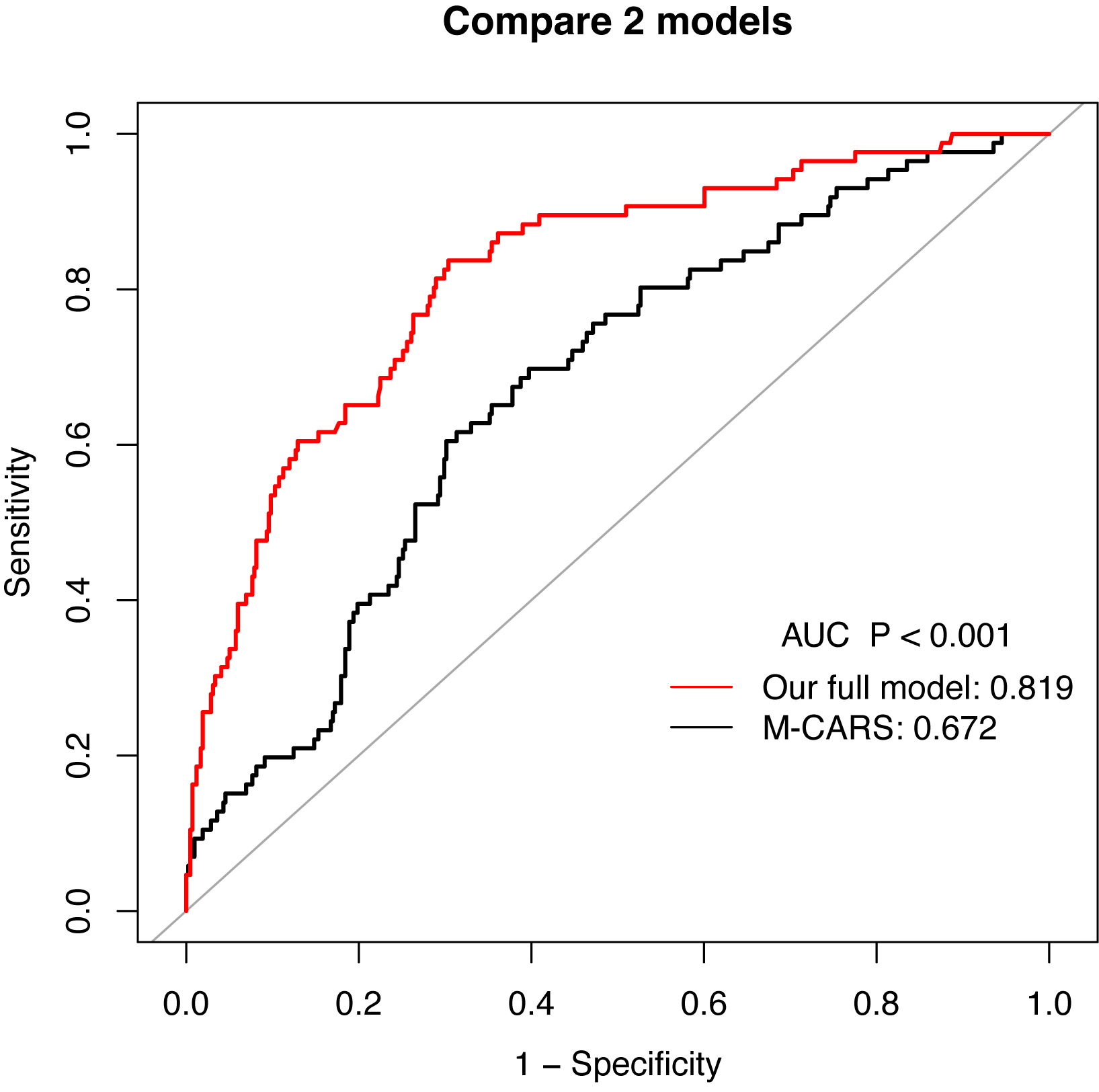


**Additional file 1: Figure S5.** The AUC for the M-CARS model was 0.672, and the AUC for our full model was 0.819.


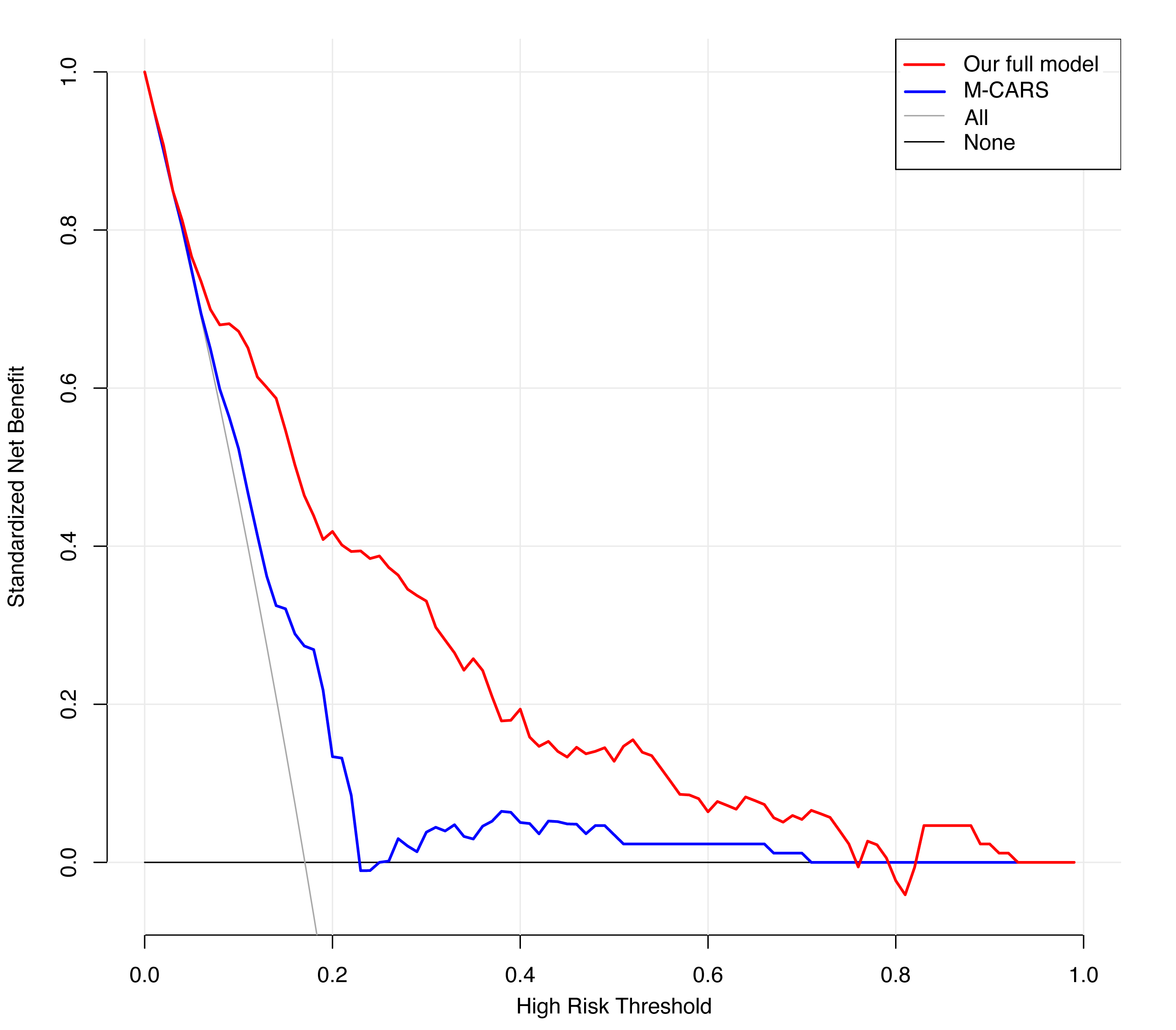


**Additional file 1: Figure S6.** The DCA curves of M-CARS model and out full model with 28-day mortality were examined.
